# Lung mesenchymal expression of *Sox9* plays a critical role in tracheal development

**DOI:** 10.1186/1741-7007-11-117

**Published:** 2013-11-25

**Authors:** Gianluca Turcatel, Nicole Rubin, Douglas B Menke, Gary Martin, Wei Shi, David Warburton

**Affiliations:** 1 Developmental Biology and Regenerative Medicine Program, Saban Research Institute, Children’s Hospital Los Angeles, Keck School of Medicine and Ostrow School of Dentistry, University of Southern California, 4661 Sunset Boulevard, Los Angeles, CA 90027, USA; 2 Department of Genetics, University of Georgia, 120 East Green Street, Athens, GA 30602, USA; 3 Department of Biosciences, Occidental College, 1600 Campus Road, Los Angeles, CA 90041, USA

**Keywords:** *Sox9*, Trachea, Lung, Cartilage, CC10, P63

## Abstract

**Background:**

Embryonic lung development is instructed by crosstalk between mesenchyme and epithelia, which results in activation of transcriptional factors, such as *Sox9*, in a temporospatial manner. *Sox9* is expressed in both distal lung epithelium and proximal lung mesenchyme. Here, we investigated the effect of lung mesenchyme-specific inducible deletion of *Sox9* during murine lung development.

**Results:**

Transgenic mice lacking *Sox9* expression were unable to breathe and died at birth, with noticeable tracheal defects. Cartilage rings were missing, and the tracheal lumen was collapsed in the mutant trachea. *In situ* hybridization showed an altered expression pattern of *Tbx4*, *Tbx5* and *Fgf10* genes and marked reduction of *Collagen2* expression in the tracheal mesenchyme. The tracheal phenotype was increasingly severe, with longer duration of deletion. Lymphatic vasculature was underdeveloped in the mutant trachea: Prox1, Lyve1, and Vegfr3 were decreased after *Sox9* knockout. We also found that compared with normal tracheal epithelium, the mutant tracheal epithelium had an altered morphology with fewer P63-positive cells and more CC10-positive cells, fewer goblet cells, and downregulation of surfactant proteins A and C.

**Conclusion:**

The appropriate temporospatial expression of *Sox9* in lung mesenchyme is necessary for appropriate tracheal cartilage formation, lymphatic vasculature system development, and epithelial differentiation. We uncovered a novel mechanism of lung epithelium differentiation: tracheal cartilage rings instruct the tracheal epithelium to differentiate properly during embryonic development. Thus, besides having a mechanical function, tracheal cartilage also appears to be a local signaling structure in the embryonic lung.

## Background

The development of the respiratory system represents an evolutionary hallmark that allowed vertebrates to survive on land utilizing air as a source of oxygen. Abnormal development of the respiratory system in humans is associated with multiple disorders such as tracheal/bronchial atresia, tracheo-esophageal fistula, bronchogenic cysts, pulmonary/lobar atresia, and pulmonary hypoplasia [1].

In the embryonic trachea, the endoderm differentiates into a ciliated pseudo-stratified epithelium, which includes basal P63+ cells, Clara cells, neuroendocrine cells, and ciliated cells. The ventral mesenchyme gradually matures to create C-shaped cartilage rings, while the dorsal mesenchyme forms contractile cells comprising the trachealis smooth muscle, which defines the posterior boundary between the trachea and esophagus. Tracheal wall malformation can be due to congenital or acquired abnormalities. The congenital forms are related to immature tracheal cartilage, whereas the more common acquired forms occur as a consequence of compression damage or degeneration of the tracheal cartilage, such as in vascular sling anomalies [2]. Tracheomalacia is a condition described as causing excessive expiratory collapse of the trachea, resulting from weakness of the supporting structures of tracheal and bronchial walls, leading to symptoms of airway obstruction [2,3].

*β-catenin*, *Bmp4*, *and Shh* are previously well-characterized regulators of cartilage formation, which may activate the *Sox9* gene in mesenchymal cells to induce their differentiation into chondrocytes. Members of the fibroblast growth factor superfamily, including *Fgf4*, *Fgf8*, and *Fgf10*, are also key regulators of cartilage formation [4,5]. *Fgf18* regulates proliferation and spatial localization of the chondrocytes in the developing upper respiratory tract [6]. *Tbx4* and *Tbx5* double knockout in lung resulted in defects of cartilage formation in mouse trachea and in altered lung branching, with *Tbx4* and *Tbx5* acting upstream of *Sox9* in tracheal cartilage development [7].

Here, we used an inducible *Tbx4* gene-driven system to knock out the *Sox9* gene specifically in lung mesenchyme [8], in order to elucidate more fully its function within the mesenchyme during lung development. Mesenchymal *Sox9* knockout had no affect on distal lung branching, on formation of smooth muscle around the bronchi, or on the trachealis smooth muscle, whereas tracheal cartilage formation was completely inhibited. *In situ* hybridization revealed that in the *Sox9* null trachea, the characteristic *Tbx4*, *Tbx5*, and *Fgf10* expression pattern was lost or severely disrupted.

Mutant *Sox9* mouse tracheal epithelium was also altered: cells had a higher number of cytoplasmic vacuoles, with fewer basal cells (P63+ cells) and more Clara cells (CC10+ cells). Mutant tracheal epithelium also had fewer goblet cells compared with wild-type trachea. Surfactant proteins A and C were also downregulated. Finally, tracheal lymphatic vessels were significantly underdeveloped after the knockout of mesenchymal *Sox9*. In conclusion, mesenchymal *Sox9*-expressing cells cover a critical developmental role in the formation of tracheal cartilage rings, and in the appropriate differentiation of lung tracheal epithelium and lymphatic system.

## Results

### *Sox9* expression during lung development

**Figure 1:**
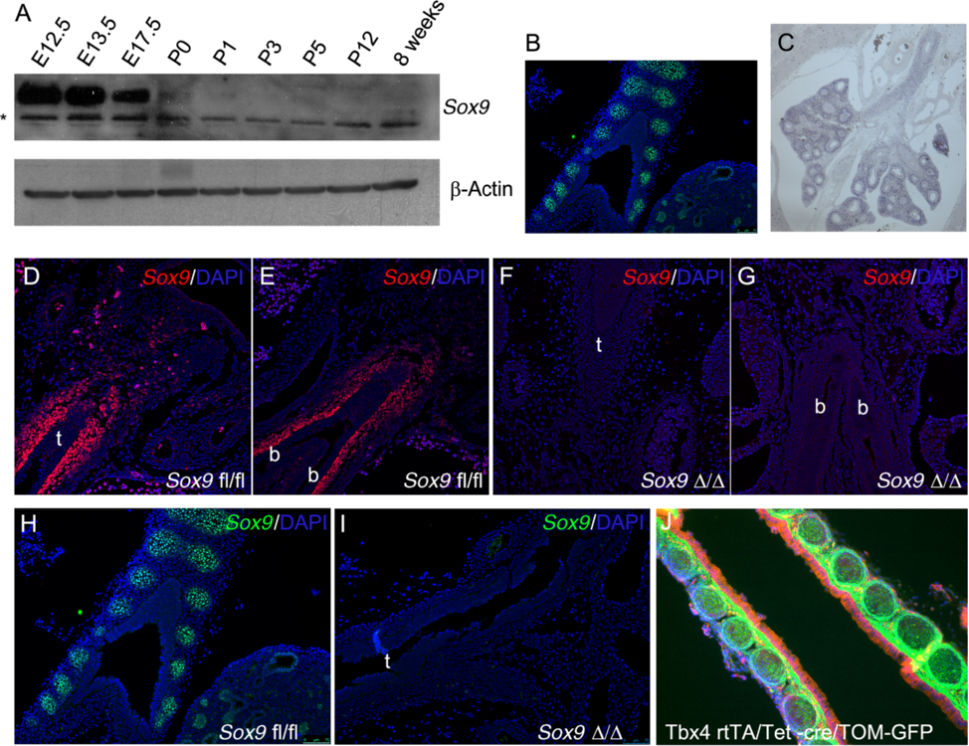
**Expression of *Sox9 *during lung development. (A)** Western blot analysis of *Sox9* protein expression during lung development at different stages (*non-specific bands). **(B)***Sox9* protein expression at E15.5 stage: immunofluorescence localization of *Sox9* showed expression of *Sox9* by tracheal immature cartilage and distal lung epithelium. **(C)** Expression pattern of endogenous *Tbx4* gene in E15.5 lung: *Tbx4* is expressed by tracheal mesenchyme and by most, but not all of the distal lung mesenchyme. **(D-I)***Sox9* immunostaining, showing effective deletion of mesenchyme *Sox-9* expression in the mutant lungs at embryonic day **(D-G)** E12.5 and **(H, I)** E15.5 stage. **(J)** Cell tracing using Tbx4-rtTA line. Tbx4-rtTA/Tet-on-cre males were bred with mT/mG females. E7.5 staged pregnant females were fed with doxocycline, and E18.5 embryos were collected. Lung epithelial cells expressed red fluorescent protein, while the mesenchyme cells are green because they are derived from *Tbx4*-expressing cells (t, trachea; b, bronchus).

We used western blot to analyze the expression of *Sox9* in mouse lung during development. *Sox9* protein expression level is high in embryonic lungs but drastically decreases in mouse lung after birth. Adult mouse lung does not express *Sox9* protein at all (Figure 1A). At embryonic day (E)12.5, *Sox9* is expressed by distal lung epithelial cells and by a population of mesenchymal cells surrounding the trachea, bronchi, and bronchioles. At E15.5, mesenchymal cells expressing *Sox9* cells are condensed at the area of the future cartilage rings, while epithelial cells expressing *Sox9* are localized in the distal lung epithelium (Figure 1B). To specifically knock out *Sox9* in mesenchymal cells, we used a Tbx4-rtTA/Tet-On-Cre system, in which rtTA gene expression is regulated by the mesenchymal specific enhancer of the *Tbx4* gene [8,9]. *Tbx4* is expressed by tracheal mesenchyme and most of the lung distal mesenchyme (Figure 1C). *Sox9* is efficiently deleted in the *Tbx4*-rtTA/Tet-On-Cre/*Sox9*^fl/fl^ at E12.5 and E15.5 stage (Figure 1D-I).

### Mesenchymal *Sox9* knockout mice showed an absence of tracheal cartilage rings

**Figure 2:**
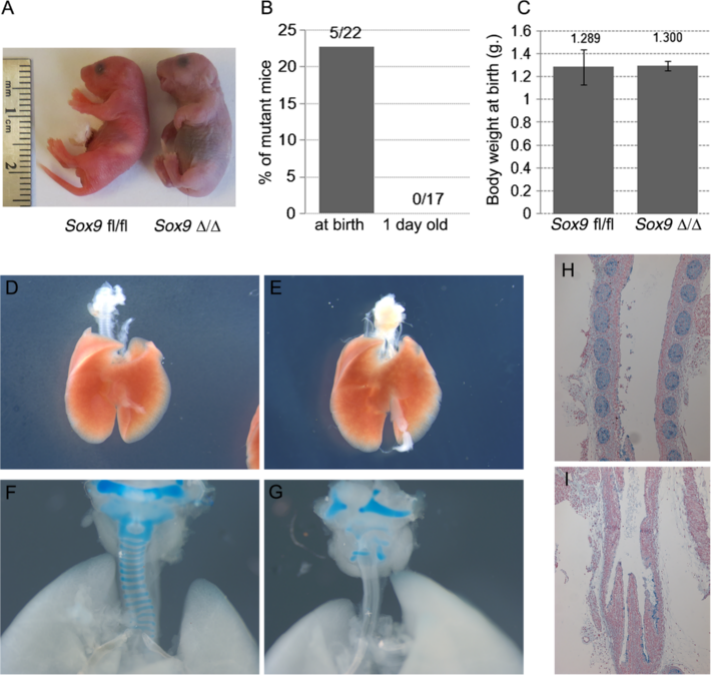
**Lung phenotype after mesenchymal *Sox9 *knockout. (A)** Pups were collected at birth. Mutant pups died less than an hour after birth, and showed gasping, retractions, and cyanosis. **(B)** Mutant pups did not survive post-natally. **(C)** Despite the respiratory mortality phenotype, no change in weight at birth was observed between the wild-type pups and the mutant pups. **(D-I)** Samples of lung at embryonic day **(E)** 18.5 were collected and analyzed. **(E)***Sox9*^Δ/Δ^ did not show obvious altered lung branching compared with **(D)** the control. **(F, G)** Alcian blue staining of **(F)** E18.5 wild-type and **(G)** mutant lungs: normal tracheal rings were observed in the control normal lung, *Sox9* knockout trachea revealed no tracheal rings. **(H, I)** Transverse section of E18.5 trachea was stained with Alcian blue. **(H)** Wild-type and **(I)** mutant mice.

**Figure 3:**
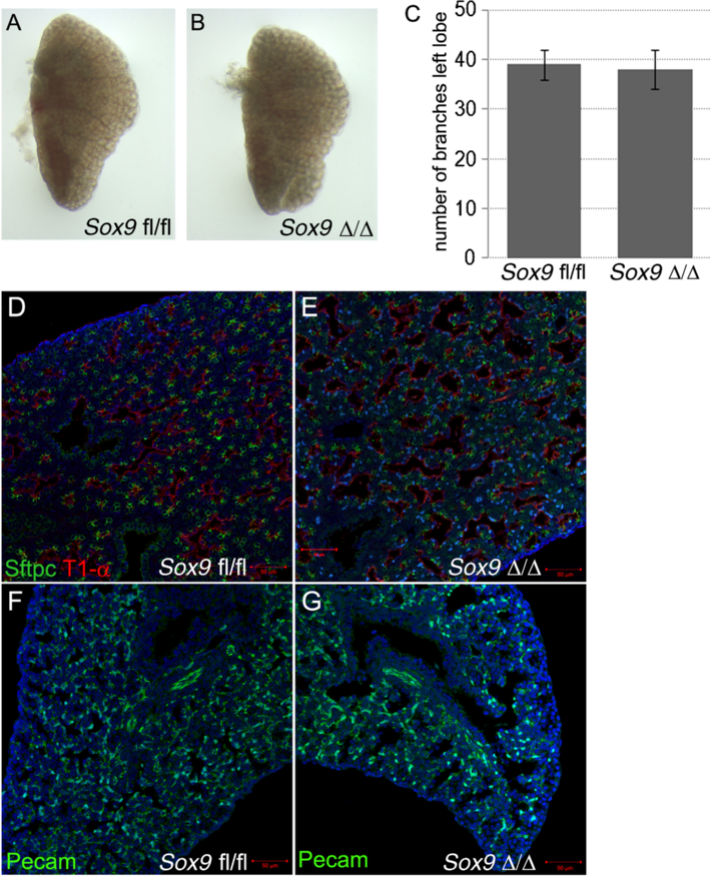
**Mesenchymal *Sox9 *deletion did not alter lung branching. (A, C)** Left lobes of **(A)** wild-type and **(B)** mutant lung at embryonic day **(E) **15.5. Terminal branches were counted, and no difference in branching morphogenesis was identified. **(D-G)** Immunostaining for Sftpc, T1-αc, and Pecam in E18.5 lungs confirmed that distal lung epithelium and mesenchyme differentiation were not altered by *Sox9* deletion in the lung mesenchyme.

Perl reported that epithelial *Sox9* deletion did not affect lung development [10]. However, more recently, Chang showed that *Sox9* deletion in lung epithelium resulted in a smaller lung, with fewer and dilated airway branches [11]. It is possible that the use of different mouse genetic backgrounds is the cause of these contrasting results. The role of mesenchymal *Sox9* expression is unknown. In our study, transgenic mice lacking mesenchymal *Sox9* expression were born at the expected mendelian frequency and appeared normal at birth, but rapidly became cyanotic and showed marked signs of respiratory obstruction including gasping and retractions (Figure 2A); moreover, not a single transgenic mouse lived more than a few hours after birth (Figure 2B). Despite the lethal respiratory obstruction phenotype, no difference in weight at birth was observed (Figure 2C). At both E15.5 and E18.5, embryonic lung branching did not appear to be affected by lack of mesenchymal *Sox9* expression (Figure 2D,E; see Additional file 1: Figure S1A-D). However, transgenic embryos were missing cartilage rings around the trachea and bronchi. (Figure 2F-I; see Additional file 1: Figure S1E,F). Transverse sections of transgenic embryo E18.5 tracheas showed collapsed airway lumen, and a shape and appearance similar to esophagus; however, the esophagus was still present. In 8% of the embryos, rudimentary cartilage spots developed on the ventral side of the trachea (see Additional file 2: Figure S2B black arrowheads). These spots were still expressing *Sox9* protein, indicating the existence of another unique population of *Sox9*+ cells that either did not continue to express the *Tbx4* gene or did not express the transgene, or expressed the transgene, but did not recombine (see Additional file 2: Figure S2C). Lung branching was not affected by lack of mesenchyme *Sox9* expression (Figure 3A-C), and differentiation of distal lung epithelium and mesenchyme appeared to be normal in the mutants (Figure 3D-G).

### The tracheal phenotype depends on the timing of doxycycline exposure

**Figure 4:**
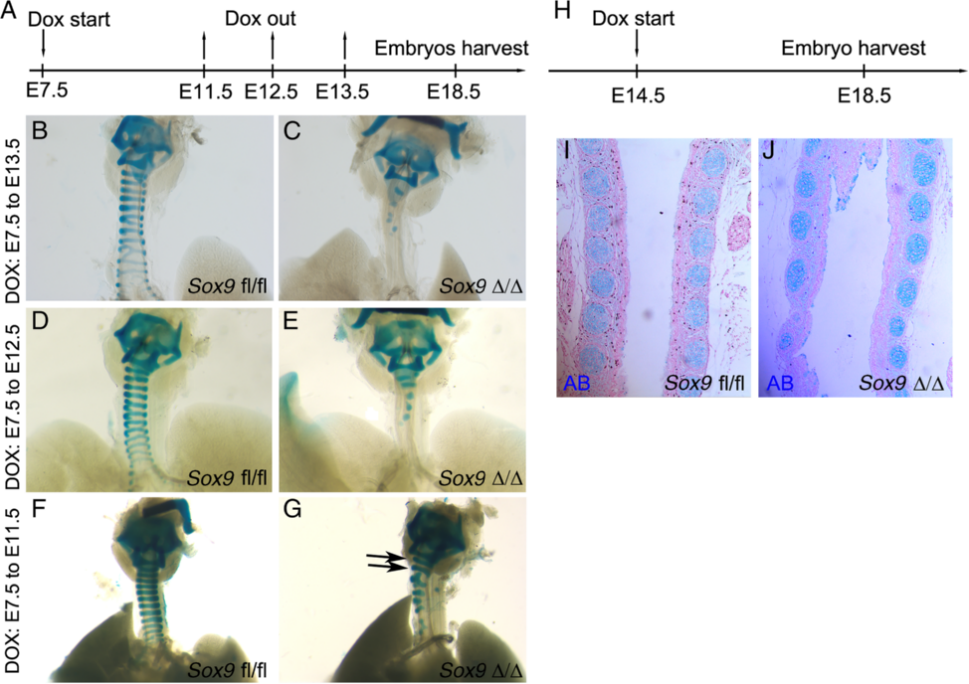
**Doxycycline induction time length affects the severity of trachea phenotype in the *Sox9 *knockout lungs. (A)** Time-plugged female mice were fed with doxycycline for different time periods, and lungs were collected at embryonic day **(E)** 18.5. **(B-G)** Doxycycline induction from **(B, C)** E7.5 to E13.5, **(D, E)** E7.5 to E12.5, and **(F, G)** E7.5 to E11.5. **(C, E, G)** The severity of the phenotype in the transgenic embryos was inversely correlated with the time length of induction. **(B, D, F)** No changes were observed in the wild-type lung tracheas. **(H-J)** E14.5 staged pregnant females were fed with doxycycline. Embryos were harvested at E18.5, and the tissues fixed and stained with Alcian blue to show the cartilage rings. **(D)** Normal cartilage rings developed in the trachea of the *Sox9*^Δ/Δ^ embryos.

The observed phenotype (Figure 2; see Additional file 1: Figure S1) was obtained by knocking out the *Sox9* gene in the mesenchyme of the lung, starting from day E7.5 to the day of collection of the embryos (E18.5). We then exposed pregnant time-mated females to different durations of doxycycline induction to determine the relation between phenotype and doxycycline induction (Figure 4A). Shorter exposure to doxycycline resulted in a milder phenotype (Figure 4B-G), as embryos exposed to doxycycline for shorter time periods developed some of the more distal cartilage rings. If doxycycline was removed from the food at E11.5, the two most proximal cartilage rings were fully formed (Figure 4F,G, black arrows). *Sox9* knockout in E14.5 embryos did not induce any abnormalities in the cartilage development, as Tbx4 and *Sox9* protein are not co-expressed in the tracheal mesenchyme at that stage (Figure 4H-J).

### Expression pattern of Tbx4, Tbx5, and Fgf10 is altered in *Sox9* mutant trachea

**Figure 5:**
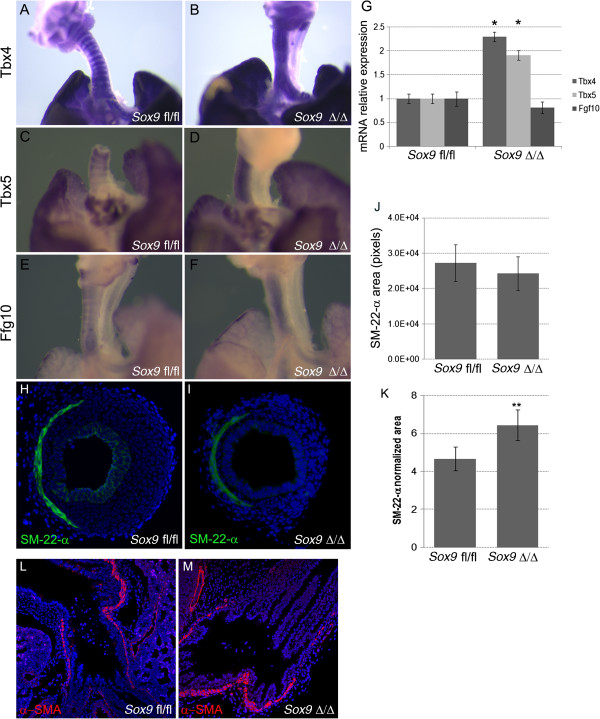
***Tbx4*, *Tbx5*, and *Fgf10 in situ *hybridization results.***In situ* hybridization for **(A, B)***Tbx4* and **(C, D)***Tbx5* was performed on lung at embryonic day **(E)** 15.5 to identify changes in expression patterns. Tbx4 andTbx5 were expressed by the mesenchyme surrounding the tracheal rings in the normal control embryonic lung tracheas **(A, C)**, whereas *Sox9* knockout lungs displayed diffuse expression of **(B)***Tbx4* and **(D)***Tbx5* along all the tracheal mesenchyme. **(E, F)** The *Fgf10* expression pattern was lost in the *Sox9*^Δ/Δ^ trachea. **(G)** Real time RT-qPCR PCR was used to determine whether expression of *Tbx4* and *Tbx5* mRNA was increased in the mutant mouse trachea versus wild-type mouse trachea (*P < 0.05). *Fgf10* expression was not significantly different between by *Sox9*^Δ/Δ^ vs. the *Sox9*^*fl/fl*^ tracheas. **(H-K)** Trachealis smooth muscle cells did not change after *Sox9* knockout. Staining for Sm-22-α was used to identify the trachealis smooth muscle cells on transverse sections of normal and transgenic mouse tracheas. **(J, K)** Even though the absolute area of trachealis smooth muscle cells did not change, the Sm-22-α relative area was significantly higher in the *Sox9*^Δ/Δ^**(J)** compared with **(K)** the *Sox9*^*fl/fl*^ trachea, because of the smaller transversal area of the mutant trachea. **P = 0.015. Values are mean ± SD, n = 5. **(L, M)**. Staining for α-smooth muscle actin (α-SMA) was used to highlight smooth muscle cells. *Sox9* deletion did not affect lung smooth muscle cell proliferation or differentiation.

We next determined whether the expression pattern of genes known to be involved in tracheal cartilage ring formation was altered in the mutant trachea. *Tbx4* and *Tbx5* are expressed between the tracheal cartilage rings in normal mouse trachea [6]. *In situ* hybridization on E15.5 embryonic lung tissue revealed that *Tbx4* and *Tbx5* expression patterning was lost in the mutant trachea (Figure 5A-D). *Tbx4* and *Tbx5* were expressed uniformly by all the mesenchyme lining the trachea (Figure 5B,D), instead of there being an alternating striped pattern as observed in the control trachea (Figure 5A,C). Using real-time reverse transcription quantitative PCR (RT-qPCR) on RNA extracted from E15.5 tracheas, we observed increased expression of *Tbx4* and *Tbx5* in the mutant trachea versus the control trachea (Figure 5G). *Fgf10* is another known key regulator of tracheal cartilage development. The *Fgf10* expression pattern in the E15.5 stage trachea normally delimits the primitive cartilage rings; however, this specific expression pattern was lost after *Sox9* knockout, and *Fgf10* was expressed by all the tracheal mesenchyme (Figure 5E,F). We observed a trend towards decreased *Fgf10* mRNA expression in the mutant trachea but it was not statistically significant.

### Trachealis contractile muscle pattern formation and bronchial smooth muscle cells are not affected by *Sox9* knockout

*The Tbx4*, *Tbx5*, and *Fgf10* expression patterns were lost in *Sox9* mutants. This observation led us to investigate whether the *Sox9* gene is also necessary for patterning the SM-22-α + cells, which are located at the junction between the trachea and esophagus (dorsal trachea), and form the trachealis muscle. Because *Sox9*-expressing cells are located at the ventral region of the trachea, *Sm-22-α* and *Sox9* expression seems to be mutually exclusive, suggesting that expression of *Sox9* is limiting the expression of *Sm-22-α* to the dorsal area of the trachea, and *vice versa*.

However, we found that *Sox9* expression is not involved in patterning the expression of *Sm-22-α*-expressing cells in the trachea (Figure 5F-K). In fact, the contractile lunar-shaped ring of Sm-22-α + cells was still present and localized in the dorsal area of the mutant trachea. The absolute area covered by Sm22-α + cells did not change significantly in the mutant trachea versus the wild-type trachea (Figure 5J). We also analyzed whether the *Sox9* gene was involved in the proliferation of bronchial smooth muscle cells; however, we did not observe any obvious change in smooth muscle cells lining the bronchi of mutant mouse lung (Figure 5L,M).

### Tracheal epithelium is altered after *Sox9* knockout

**Figure 6:**
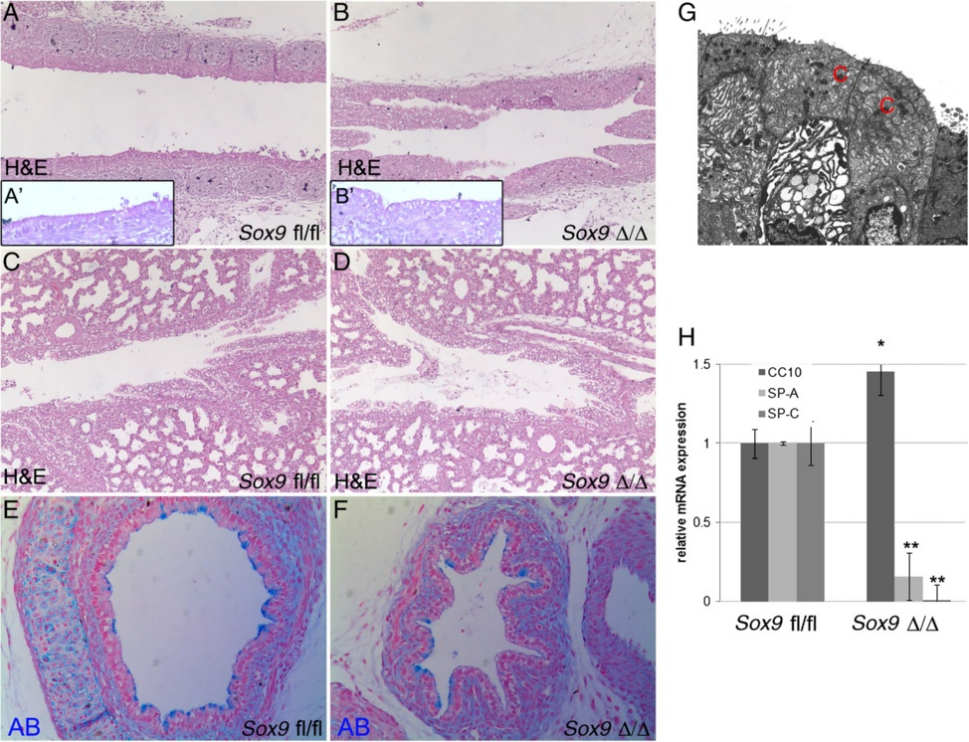
**Lung tracheal epithelium is altered in *Sox9 *knockout mice. (A-B)** Hematoxylin and eosin (H&E) staining of embryonic day **(E)** 18.5 tracheal sections, showing altered morphology of epithelial cells in **(B)** the mutant trachea compared with **(A)** the wild-type (**A′** and **B′** are high magnification inserts of **A** and **B** respectively). **(C, D)** Intralobar sections of main bronchi stained with H&E. No obvious differences are seen between **(C)** the wild-type and **(D)** mutant intralobar bronchia. **(E, F)** Transverse sections of **(E)** wild-type and **(F)** mutant mouse tracheas stained with Alcian blue. Mutant tracheas are collapsed because of the lack of structural support normally given by the cartilage rings. **(G)** Electron microscopy of epithelial cells lining the mutant tracheal revealed the presence of Clara cells. **(H)** Real time RT-qPCR analysis at E18.5 altered expression of *Sftpc*, *Sftpa*, and *CC-10* genes. *P < 0.05, **P < 0.01. Values are mean ± SD.

**Figure 7:**
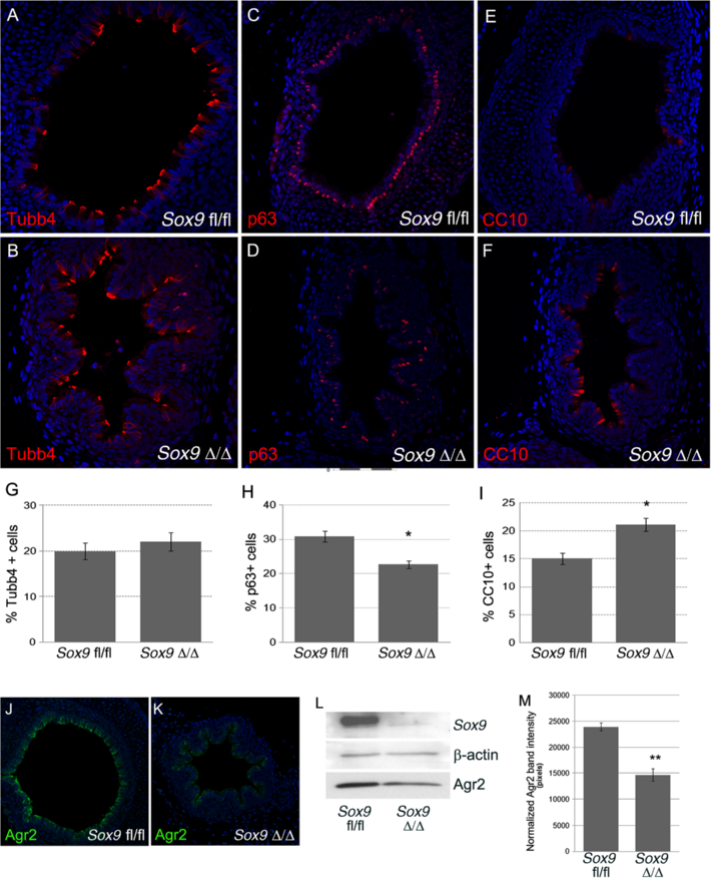
**Basal cell numbers are decreased and Clara cell numbers are increased in the mutant mouse trachea. (A-B)** Ciliated cell number was not altered in *Sox9* knockout mouse tracheas. Staining for β-tubulin-IV on transverse section of wild-type and mutant mouse trachea was used to determine change in ciliated cell numbers. **(G)** The number of ciliated cells did not change in the mutant mouse trachea versus the wild-type mouse trachea. **(C, D)** Staining for P63 was used to confirm that the number of basal cells is decreased in the mutant mouse trachea. **(E, F)** CC10 marker staining was used to highlight the increase in Clara cell number in *Sox9* mutant mouse trachea. **(G-I)** Cell count statistics. *P <0.03. Values are mean ± SD, n = 4. **(J-M)***Sox9*^Δ/Δ^ tracheas had a reduced number of goblet cells. **(J, K)** Immuno-fluorescence staining and **(L)** western blot for Agr2 protein were used to determine change in goblet cells after *Sox9* knockout. *Sox9* knockout resulted in reduced production of Agr2 by the tracheal epithelium, suggesting reduced numbers of goblet cells in the *Sox9*^Δ/Δ^ trachea. **(M)** Intensity analysis of Western blot bands. **P < 0.05. Values are mean ± SD.

Mice lacking *Sox9* in the lung mesenchyme died at birth, due to an inability to breathe because of the collapsed trachea. No change in smooth muscle cell numbers or patterning was observed. However, the epithelium lining the trachea was abnormal; epithelial cells in the mutant trachea had larger and more numerous vacuoles (Figure 6B,F). Electron microscopy revealed that some of those cells looked like Clara cells (Figure 6G). Real-time RT-qPCR was used to quantify change of expression of lung-relevant genes in mutant mouse trachea. Expression of the surfactant protein genes *Sftp-a* and *Sftp-c* mRNA was reduced in the *Sox9*^Δ/Δ^ mice, and *CC10* mRNA was slightly overexpressed in the mutant trachea (Figure 6H), indicating altered differentiation of tracheal epithelium. The number of ciliated cells did not change after mesenchymal deletion of the *Sox9* gene (Figure 7A,B,G). However, at E18.5, the number of tracheal basal cells (P63+) decreased (Figure 7C,D,H) and Clara cells (CC10+) (Figure 7E,F,I) increased in the mutant trachea versus the control trachea. E15.5 *Sox9*^*fl*/*fl*^ and *Sox9*^∆/∆^ tracheas showed a similar number of P63+ cells (see Additional file 3: Figure S3). Alcian blue staining of transverse sections of trachea revealed decreased mucin production by the mutant tracheal epithelium (Figure 6E,F). Agr2 protein, a marker of lung goblet cells, was downregulated in the tracheal epithelium of the *Sox9*^*Δ/Δ*^ embryos compared with the *Sox9*^*fl/fl*^ embryos (Figure 7J-M). However, expression of Sox2 and Foxp1, key regulators of lung epithelium differentiation, was unaltered after *Sox9* knock out (see Additional file 4: Figure S4).

**Figure 8:**
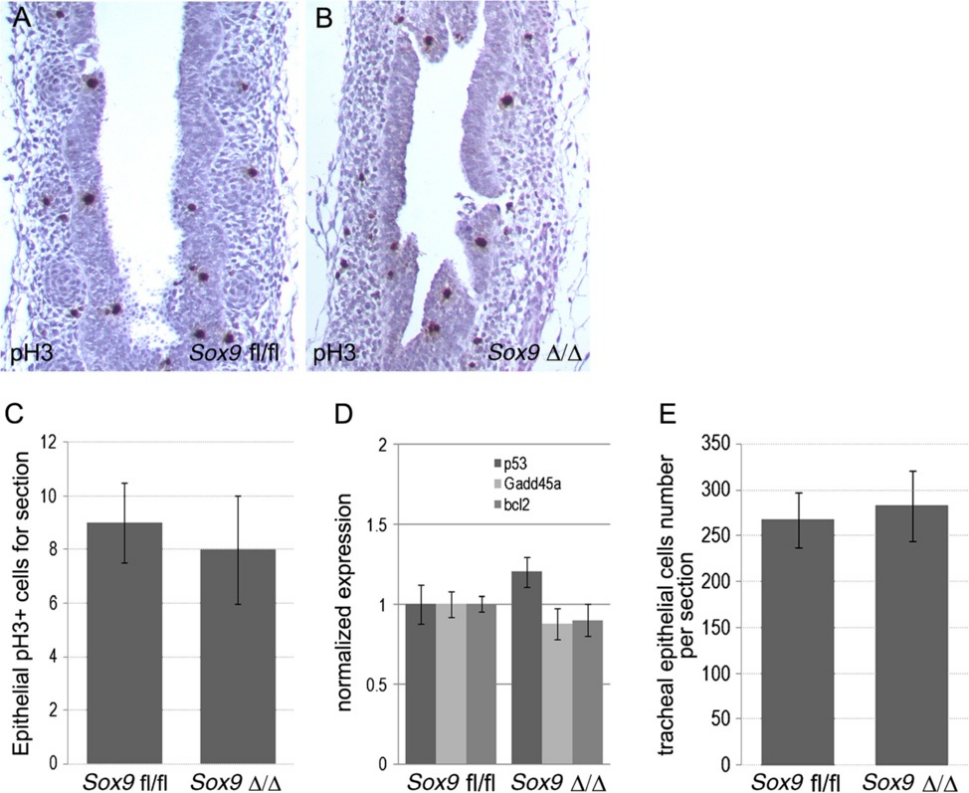
**Tracheal epithelial differentiation and apoptosis was not altered by lack of *Sox9 *gene. (A, B)** Phospho-histone-3 (pH3) staining on longitudinal sections of **(A)** wild-type and **(B)** mutant tracheas at embryonic day **(E)** 15.5 did not reveal any change in epithelial cell proliferation. **(C)** Number of pH3-positive cells per section was similar between wild-type and mutant mouse trachea. **(D)** Real-time PCR for the apoptotic markers *p53*, *Bcl2*, and *Gadd45a* on total RNA extracted from E15.5 wild-type and mutant tracheas indicated no change in apoptosis. **(E)** Tracheal epithelial cells were counted on transverse sections of *Sox9*^*fl/fl*^ and *Sox9*^Δ/Δ^ E18.5 tracheas, revealing no change in cell number between the two groups. Values are mean ± SD.

Despite the altered differentiation, proliferation of tracheal epithelium was not affected by lack of mesenchymal *Sox9* expression, No change in phospho-histone-3 (pH3) and total cell number per tracheal transversal section was found (Figure 8A-E).

### Tracheal lymphatic system was altered in *Sox9*^Δ/Δ^ trachea

**Figure 9:**
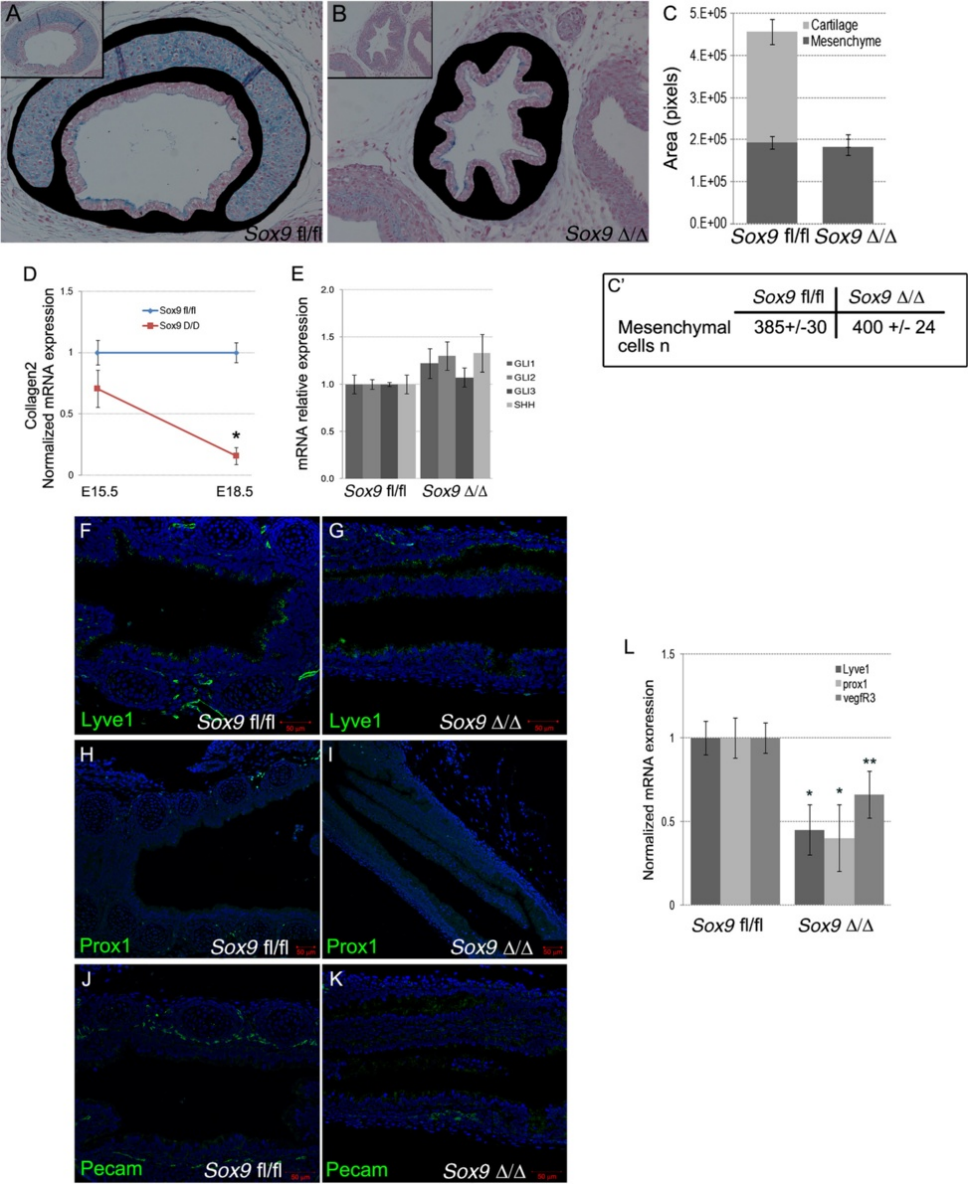
**Lymphatic vascular system was affected by *Sox9 *deletion. (A-C)** Tracheal mesenchyme area quantification analysis did not reveal changes between the wild-type and mutant tracheas (tracheal mesenchyme was stained in black). **(C′)** The number of mesenchymal cells per section was similar between wild-type and mutant trachea. **(D, E)** Real time RT-qPCR for *Collagen2*, *Gli1*, and *Shh* on mRNA from wild-type and mutant tracheas at embryonic day **(E)** 15.5. *Col2* expression was decreased in the *Sox9*^Δ/Δ^ trachea starting at E15.5 stage. No statistically significant differences in expression of *Shh*, *Gli1*, *Gli2*, or *Gli3* was observed between *Sox9*^*fl/fl*^ and *Sox9*^*Δ/Δ*^. **(F-L)** The lymphatic vascular system was underdeveloped in the mutant trachea. Immunostaining of the lymphatic markers **(F, G)** Lyve1, **(H, I)** Prox1, and **(J, K)** Pecam revealed that *Sox9* deletion resulted in impaired lymphatic vessel formation. **(L)** Real time RT-qPCR for *Prox*, *Lyve1*, and *Vegfr3* confirmed that the lymphatic vascular system was not properly developed in the *Sox9* knockout tracheas. *P < 0.05, **P = 0.08.

We quantified the area covered by lung mesenchyme (excluding the cartilage) and the mesenchyme cell number on transverse sections of E18.5 wild-type and mutant tracheas. Despite the somewhat misleading visual appearance, both the mesenchyme cell numbers and the surface covered by them were the same in the *Sox9*^*fl/fl*^ and *Sox9*^Δ/Δ^ trachea (Figure 9A-C,C′). *Prox1* and *Lyve1* are key markers of lymphatic vessel development [12]. *Prox1* also controls neuronal progenitor cell differentiation to maintain the required balance between different cell types [13]. We found that the tracheal lymphatic system was significantly underdeveloped in the mutant *Sox9* embryos, as shown by reduced immunostaining of Prox1, Lyve1, and Pecam (Figure 9F-K). In addition, the, mRNA level of *Prox1*, *Lyve1*, and *Vegfr3* was reduced in the *Sox9*^Δ/Δ^ tracheas (Figure 9l).

## Discussion

Normal lung branching is achieved by tightly regulated crosstalk between mesenchymal and epithelial compartments. Specific growth factors are released from one compartment to activate expression of genes in the other compartment, and *vice versa*[14,15]. *Sox9* is a multifunctional transcriptional factor expressed in both embryonic lung mesenchyme and epithelium. Perl *et al*. reported that deletion of *Sox9* gene in embryonic lung epithelium did not affect lung development; mice lacking *Sox9* in the lung epithelium were viable and fertile, and did not show any differences from their control littermate, even when exposed to hyperoxia-induced injury [10]. However, more recently, Chang *et al*. showed that *Sox9* is necessary for proper lung branching [11]. *Sox9* is also a key regulator of embryonic kidney epithelial branching [16]. These contrasting reports on *Sox9* regulation of lung epithelial lung branching may be due to the use of different mouse genetic backgrounds by Perl and Chang. Here, we focused on the role of the mesenchymal *Sox9*-expressing cells, which are localized around the trachea and main bronchi. By using an inducible doxycycline-regulated system, driven by part of the *Tbx4* gene promoter [8], we were able to delete *Sox9* expression specifically in the mesenchymal compartment of the murine lung. Our collaborator Shi [9] developed the Tbx4-rtTA transgenic mouse line and in the present study, we showed that this mouse line specifically deleted *Sox9* in lung mesenchyme (Figure 1D-I) and likewise activated a green fluorescent protein (GFP) reporter gene in lung mesenchyme (Figure 1J).

Mice lacking *Sox9* did not develop tracheal cartilage rings, were unable to breathe because of tracheal collapse, and died shortly after birth. Transgenic mice appeared normal at birth, but then rapidly showed clear evidence of cyanosis (Figure 2A), and not a single transgenic pup lived more than a few hours after birth (Figure 2B), despite the birth weights of the mutants being similar to those of wild-type mice (Figure 2C). These data suggest that lack of mesenchymal *Sox9* in embryonic lung is not compatible with life.

Arora *et al*. obtained a similar but milder tracheal cartilage phenotype in transgenic mice missing both the *Tbx4* and *Tbx5* genes; some cartilage trachea rings still developed, but they were smaller and had an abnormal shape [7]. A *Tbx4* and *Tbx5* combined deletion also resulted in altered lung branching. Data from the same laboratory also suggested that *Tbx4* and *Tbx5* are upstream regulatory genes of *Sox9* in the embryonic lung mesenchyme [7].

Lung mesenchyme *Sox9* knockout mice did not show an obvious phenotype on lung branching; the number of terminal branches of the left lobe of E15.5 lungs was not different between the wild-type and mutant embryos (Figure 3A-C). Moreover, differentiation of distal lung epithelium and mesenchyme was not affected by lack of *Sox9* mesenchymal expression at E18.5 (Figure 3D-G). Thus, *Sox9* deletion appears to affect only tracheal development.

In our mesenchyme-specific *Sox9* knockout model, *Tbx4* and *Tbx5* expression was enhanced in the trachea. This indicates the existence of a complex feedback from *Tbx4/5* to *Sox9* and *vice versa*. Thus, *Tbx4/5* gene expression may have been upregulated, and may thus have partially compensated for the lost *Sox9* expression in our mutant.

Moreover, *Sox9* knockout mice also had an altered *Tbx4/5* pattern of expression. In normal lung trachea, the *Tbx4* and *Tbx5* genes are expressed by the mesenchyme in the intervals between the developing cartilage rings. Thus, the *Tbx4/5* genes and the *Sox9* gene are normally mutually exclusive in the tracheal mesenchyme at E15.5. When the *Sox9* gene was knocked out, the *Tbx4/5* genes were uniformly expressed by all the mesenchyme surrounding the trachea. A similar result was obtained with *Fgf10* pattern expression. At E15.5, *Fgf10* is normally expressed by the developing cartilage rings in the trachea. Inactivation of *Sox9* resulted in the disruption of the normal *Fgf10* spatial expression pattern; *Fgf10* was now expressed by all the mesenchyme surrounding the trachea. Quantification of the mRNA of *Fgf10* did show a small trend towards a decrease in mRNA level, but this did not reach statistical significance.

Taken together, these data clearly indicate that the *Sox9* gene has a key role in patterning the expression of *Tbx4/5* and *Fgf10* in the embryonic trachea. Genes linking *Sox9* expression to *Tbx4/5* and *Fgf10* gene expression pattern will be the subject of future studies.

The Tbx4-rtTA inducible system allowed us to closely regulate the timing of *Sox9* deletion. We took advantage of this to investigate how the length of time of doxycycline induction relates to the severity of the phenotype. We found that shorter induction resulted in a milder phenotype. If the doxycycline was removed from the food at E11.5, the two most distal cartilage rings formed fully (Figure 4G), but in these mice, the *Tbx4* gene was expressed by all the lung mesenchyme [7], which implies that all lung mesenchymal cells in these mice have a non-functional *Sox9* gene at that stage. There are several possible explanations for these results. It could be that mesenchymal *Sox9*-positive cells are continuously migrating from the laryngeal region into the tracheal mesenchyme to contribute to cartilage ring formation, or that the shorter doxycycline exposure results in incomplete *Sox9* deletion. Another possible explanation is that Tbx4-rtTA is expressed in distal mesenchyme and then proceeds towards the larynx over time. Future studies involving the use of a *Sox9-ER-cre* mouse line in combination with our Tbx4-rtTA model should be able to determine which explanation is likely to be the correct one. However, considering that *Sox9* is also expressed by neural crest cells [17,18], which are ‘migratory’ cells, the hypothesis of *Sox9* continuously migrating into the lung mesenchyme seems to be the most plausible, and it will be the first hypothesis to be tested in our future studies. By E14.5 to E15.5, tracheal *Tbx4* and *Sox9* are mutually exclusively expressed, thus as predicted, knockout of the *Sox9* gene atat this later stage (E14-5 to E15-5) did not affect tracheal cartilage development (Figure 4). The ability to regulate the severity of the tracheal phenotype will allow us to tailor timed knockouts to model specific tracheal and lung disease models for future studies.

As pointed out above, mesenchymal-epithelial crosstalk is required for correct development of the embryonic lung. Therefore, anatomical or molecular alterations of one embryonic compartment such as mesenchyme may affect the correct development of the epithelia, and *vice versa*. We noticed that the tracheal epithelium was altered after mesenchymal *Sox9* knockout. Cell count analysis revealed that mutant trachea had a lower number of basal P63+ cells, and a higher number of Clara (CC10+) cells. The number of ciliated cells did not change. An immediate conclusion would be that the absence of cartilage rings around the embryonic trachea caused the tracheal epithelium to behave more similarly to the more distal intralobar bronchiolar epithelium. As the number of basal cells was not altered in the E15.5 *Sox9*^*Δ/Δ*^ trachea, we suggest that basal cell differentiation is not directly dependent on *Sox9* expression but is more likely to depend on cartilage condensation, which normally starts to occur at E15.5. Thus, we speculate that a specific growth factor signal (or signals) may be released by the cartilage rings to promote correct tracheal epithelium differentiation.

*Sox9*^*Δ/Δ*^ trachea had reduced production of Agr2, a protein that identifies goblet cells in the lung. Moreover, transcripts of both surfactant proteins A and C were reduced in the mutant trachea, further supporting the role of *Sox9* expressing cells in the differentiation of tracheal epithelium.

Despite the altered differentiation, proliferation of tracheal epithelium was not affected by lack of mesenchymal *Sox9* expression. No change in pH3 or epithelial cell number per transversal section was found (Figure 8A-E). Thus, lack of tracheal rings altered proper differentiation of tracheal epithelium but not its proliferation.

The expression pattern of *Fgf10* in *Sox9*^*Δ/Δ*^ embryonic trachea was markedly altered. Even though it was not statistically significant, *Fgf10* mRNA level was slightly decreased in the mutant trachea. Volckaert *et al*. [16] showed that *Fgf10* overexpression resulted in an increased number of basal cells and decreased number of Clara cells [19]. Because of the concordance of these and our results, we hypothesize that incorrect expression of *Fgf10* may be mostly responsible for the altered differentiation of tracheal epithelium in the *Sox9*^*Δ/Δ*^ trachea. We cannot conclude without any doubt that the epithelial phenotype is directly related to the absence of signals originating from the cartilage rings. An alternative plausible model is that the lack of mechanical support (and thus collapse) of the trachea instructs the epithelium to differentiate onto a different path. Finally, the lack of proper vasculature development (discussion below) in the mutant trachea may also contribute to the altered differentiation of the tracheal epithelium.

Gli2^–/–^,Gli3^+^/– mutants displayed esophageal atresia with tracheo-esophageal fistula and a severe lung phenotype [20]. Gli2 and Gli3 gene expression in the *Sox9*^*Δ/Δ*^ trachea did not change in our *Sox9* knockout model (Figure 9E). *Shh* and *Gli1* are also key regulators of cartilage development [21], but their expression was not altered in our *Sox9*^*Δ/Δ*^ embryonic trachea (Figure 9E). Expression of *Collagen2*, a key regulator of cartilage differentiation, was reduced after *Sox9* deletion, supporting the model in which *Sox9* acts upstream of *Collagen2* to control tracheal cartilage development.

Despite the somewhat misleading visual appearance, the numbers of mesenchymal cells and the area covered by them were not different between the *Sox9*^*fl/fl*^ and *Sox9*^*Δ/Δ*^ trachea (Figure 9A-C,C′). Thus, the apparent difference in size observed between the wild-type and mutant tracheas is caused by the lack of the cartilage rings in the *Sox9*^*Δ/Δ*^ trachea. *Pecam* is broadly expressed by vascular structures [22,23]. and its expression in the distal lung mesenchyme was assayed to investigate any alterations of distal mesenchyme lung differentiation. As we observed for the distal epithelium, the distal lung mesenchyme appeared normal in the mutant trachea. We concluded that lack of *Sox9* in the lung mesenchyme did not affect the differentiation or development of the vasculature in the distal lung mesenchyme.

The lymphatic vascular system serves to transport tissue fluid, extravasated plasma proteins, and cells back to the circulation [24,25]. *Prox1* and *Lyve1* are key regulators and markers of lymphatic vessel development. The lymphatic system was significantly underdeveloped in the mutant *Sox9* tracheas; immunostaining of *Prox1*, *Lyve1*, and *Pecam*, and mRNA level of *Prox1*, *Lyve1*, and *Vegfr3* were reduced in the *Sox9*^*Δ/Δ*^ tracheas. Thus, lack of tracheal cartilage affects appropriate tracheal lymphatic vessel development. It is also possible that an early *Sox9*-expressing mesenchymal cell population exists, and that this differentiates into lymphatic vessel cells. As *Pecam* is expressed by both blood and lymphatic vascular vessels, we speculate that mesenchymal *Sox9* expression has a broad role in the generalized vascularization of the trachea. The mechanism linking the lack of cartilage rings to the altered tracheal vascularization is under further investigation. We focused on epidermal growth factor, as it is known to be involved in lymphatic vessel modeling in the skin [26], and found it to be downregulated in the *Sox9*^*Δ/Δ*^ trachea (data not shown).

## Conclusions

**Figure 10:**
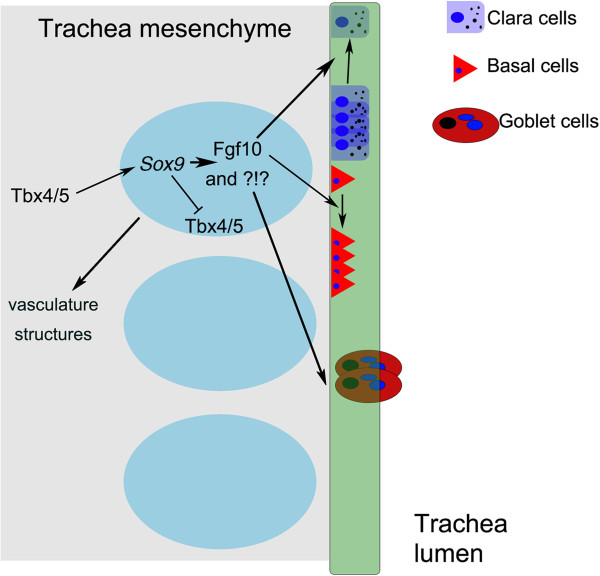
**Concept diagram of *Sox9 *function in lung development.***Sox9* acts downstream of *Tbx4/5*[6]. *Sox9* feeds back negatively on *Tbx4* and *Tbx5* in the tracheal mesenchyme. Cartilage ring formation requires functional *Sox9* expression in the tracheal mesenchyme. Lack of cartilage rings induce altered tracheal epithelium differentiation (increased Clara cells, and decreased basal and goblet cells) and defective development of tracheal vasculature structurest.

In conclusion, we have uncovered a key fundamental role of the mesenchymal *Sox9*-expressing cells in the development of the tracheal cartilage rings, which in turn induce proper tracheal epithelium differentiation and lymphatic system development (Figure 10). Thus, besides having a mechanical function, tracheal cartilage also appears to be a local signaling structure in the embryonic lung. Some pieces of this puzzle are still missing, and are currently under investigation. Nevertheless, we have identified an important mechanism by which mesenchymal *Sox9*-expressing cells instruct tracheal cartilage, epithelial, and lymphatic differentiation in a phenotype that already appears to model and may perhaps explain mechanistically some extreme cases of primary tracheomalacia in human new born infants.

The appropriate temporospatial expression of *Sox9* in lung mesenchyme is necessary for correct tracheal cartilage formation, lymphatic vasculature system development, and epithelial differentiation. Our study showed that a lack of tracheal cartilage rings resulted in increase in Clara cells and decrease in basal and goblet cells in the tracheal epithelium. We also uncovered a novel mechanism of lung epithelium differentiation; tracheal cartilage rings instruct the tracheal epithelium to differentiate properly during embryonic development. Thus, besides having a mechanical function, tracheal cartilage also appears to be a local signaling structure in the embryonic lung.

## Methods

### Ethics statement

We strictly followed the National Institute of Health guidelines for animal care and safety. The animal experimental∆ protocols were approved by the Animal Care and Use Committee at the Children’s Hospital Los Angeles (CHLA), where the vivarium is under weekly surveillance by an external chief veterinarian to check animal health. Our animal facility is approved by the AAALAC (Association for Assessment and Accreditation of Laboratory Animal Care) and USDA (The United States Department of Agriculture).

### Mice

*Sox9*^*fl/fl*^ mice were bought from Jackson Laboratories (Sacramento, CA). Tbx4-rtTA mice were kindly provided by W Shi (CHLA) [9]. The plasmid bearing the lung-specific enhancer of *Tbx4* was kindly provided by D B Menke (University of Georgia) [8]. Tet-On-Cre mice were bought from Jackson Laboratories. Mt/mG mice (stock number 007576) were bought from Jackson Laboratories. Tbx4-rtTA/Tet-on-cre mice were generated and crossed with female *Sox9*^*fl/fl*^. Timed pregnant mice were fed with doxycycline chow (rodent diet with 0.0625% doxycycline; Harlan Teklad TD01306) from E7.5 stage, unless specified.

### Alcian blue staining

Alcian blue staining was performed in accordance with standard protocols. Lungs and trachea were dissected out at different stages of life, and fixed in 95% ethanol for 1 to 2 days, stained in Alcian blue solution for 1 day, washed with 95% ethanol for 1 to 2 days, and finally clarified in 1% potassium hydroxide solution for 2 to 5 hours [5]. For Alcian blue staining on tissue sections, 5 μm thick paraffin wax-embedded sections were rehydrated, stained with 1% Alcian blue in 3% acetic acid for 30 minutes, washed in distilled water, and counterstained with Nuclear Fast Red.

### Whole-mount *in situ* hybridization

E15.5 lungs were isolated from embryos and fixed for 2 hours in 4% paraformaldehyde in phosphate-buffered saline (PBS). The samples were washed twice in PBS for 5 minutes, transferred to 70% ethanol overnight, and stored in 100% ethanol until needed. Whole-mount *in situ* hybridization was performed as previously described, with riboprobes transcribed from murine plasmid DNA templates [5].

### Immunohistochemistry and histology

Immunohistochemistry and immunofluorescence were performed on 5 μm thick paraffin wax-embedded sections, as described previously. Primary antibodies used were anti-Sm22-α, P63, *Sox9*, CC10 (Santa Cruz Biotechnology), anti-Tubb4 (BioGenex), (Developmental Studies Hybridoma Bank antibody 8.1.1), anti-Agr2 (Abgent), anti-phospho-H3, β-catenin (Cell Signaling Technology, Danvers, MA), α-SMA (Sigma-Aldrich), anti-PECAM (LifeSpan Bioscences Inc.), anti-Prox1, and Lyve1 (both Angiobio). The Alexa Fluor 488, 555, and 647 secondary antibodies were from Invitrogen.

For histological evaluation, embryos were removed from the uterine horns, and dissected free from the decidua, then the lungs were removed and fixed in 4% formalin at room temperature. After dehydration in ethanol, embryos were embedded in paraffin wax, sectioned at 5 μm thickness and stained with hematoxylin and eosin or Alcian blue.

### cDNA reverse transcription and real-time reverse transcription PCR

Total RNA was extracted from mouse tracheas using Trizol reagent, then 500 to 1,000 ng of RNA was reverse-transcribed using the IscriptcDNA synthesis kit (Bio-Rad, Hercules, CA, USA). RNA was extracted from a pool of three tracheas to get sufficient good-quality RNA for expression analysis. Real-time quantitative PCR was performed in a Lightcycler II (Roche Applied Science) using gene-specific primers (Operon, Huntsville, AL, USA) and fluorescence probes (Roche Applied Science).

### Western blot

Embryos were dissected in cold PBS, tracheas were separated from the esophagus and lysed with RIPA buffer (1 × Tris-buffered saline, 1% Nonidet P-40, 0.5% sodium deoxycholate, 0.1% SDS, 0.004% sodium azide; Sigma-Aldrich, St. Louis, MO, USA), supplied with phosphatase and protease inhibitors (phenylmethylsulfonyl fluoride (PMSF) solution, sodium orthovanadate solution, and protease inhibitor cocktail solution; Sigma-Aldrich). Three tracheas from each experimental group (wild-type and control) were combined together to achieve a sufficient amount of protein lysate. β-actin protein amount was used as loading control and measured in each western blot experiment.

### Cell count and cellular area analysis

A Leica confocal microscope (Leica) was used to capture fluorescent images. For epithelial and mesenchymal cells counts, (Figure 8C, Figure 9C), 4 μm thick tracheal transversal sections were used; nuclei were highlighted with DAPI or hematoxylin, and counted with ImageJ software.

For mesenchymal area analysis, the mesenchyme area on transversal sections of tracheas was highlighted, and pixels were counted using Photoshop software.

### Statistical analysis

The student’s t-test was used to perform statistical analysis. Every experiment was repeated at least three times unless indicated.

## Competing interests

The authors declare that they have no competing interests.

## Authors’ contributions

GT and DW conceived of the study; GT performed the experiments and participated in study design; NR and GM performed the electron microscopy studies; WS and DW designed experiments; and NR, DW, WS, and DBM helped to draft the manuscript. All authors read and approved the final manuscript.

## Supplementary Material

Additional file 1: Figure S1 Lung phenotype after *Sox9* knockout at embryonic day (E) 15.5. (A-D) Bright-field and dark-field pictures of (A, C) wild-type and (B, D) mutant *Sox9* knockout mouse lungs. (E, F) Alcian blue staining of longitudinal sections of (E) wild-type and (F) mutant *Sox9* knockout mouse trachea. C′ and D′ are high magnification pictures of C and D, respectively. Click here for file

Additional file 2: Figure S2 Incomplete phenotype in *Sox9*^*Δ/Δ*^ trachea. (A, B) A small percentage of the *Sox9*^*Δ/Δ*^ lung developed proximal rudiments of cartilage in the ventral side of trachea. (C) Transverse section of lung in (B) stained for *Sox9*. Click here for file

Additional file 3: Figure S3 Staining for P63 was used to determine changes in basal cells in E15.5 *Sox9*^*Δ/Δ*^ trachea compared with *Sox9*^*fl/fl*^ trachea. (A-C) Number of P63-positive cells in the tracheal epithelium was not affected by *Sox9* deletion at embryonic day (E) 15.5. Click here for file

Additional file 4: Figure S4 Sox2 and Foxp1 expression was not altered in *Sox9*^*Δ/Δ*^ tracheal epithelium. Immunofluorescence staining for Sox2 and Foxp1 did not show any qualitative or quantitative alterations of expression of these transcriptional factors in the mutant trachea. Click here for file
