# Supplementary figures and images for: Lung mesenchymal expression of Sox9 plays a critical role in tracheal development
Source: BMC Biol. 2013 Nov 25;11:117. doi: 10.1186/1741-7007-11-117 (PMC4222279; doi:10.1186/1741-7007-11-117)

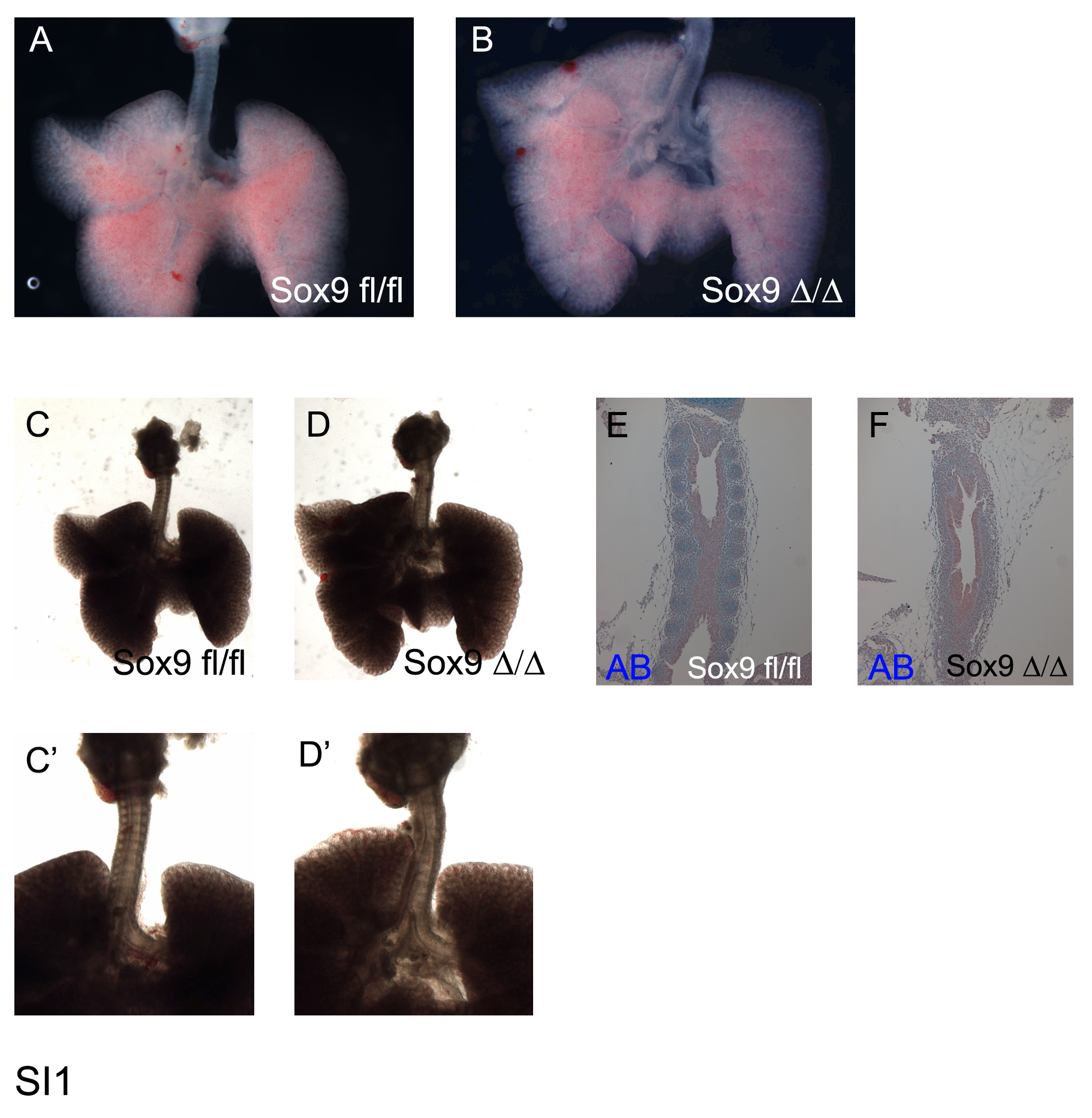

Supplement: Additional file 1: Figure S1 — Lung phenotype after Sox9 knockout at embryonic day (E) 15.5. (A-D) Bright-field and dark-field pictures of (A, C) wild-type and (B, D) mutant Sox9 knockout mouse lungs. (E, F) Alcian blue staining of longitudinal sections of (E) wild-type and (F) mutant Sox9 knockout mouse trachea. C′ and D′ are high magnification pictures of C and D, respectively. [file 1741-7007-11-117-S1.tiff]

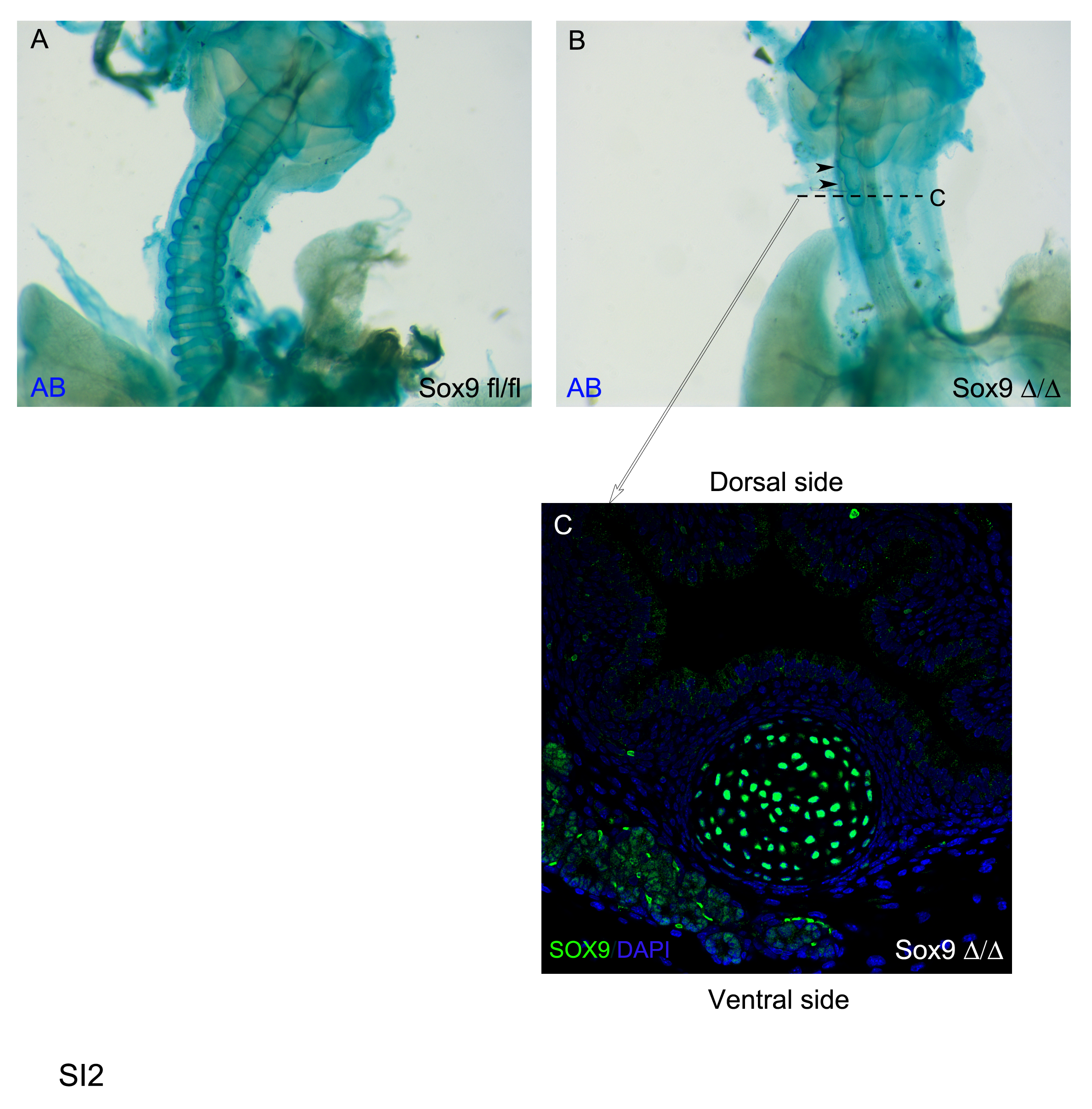

Supplement: Additional file 2: Figure S2 — Incomplete phenotype in Sox9Δ/Δ trachea. (A, B) A small percentage of the Sox9Δ/Δ lung developed proximal rudiments of cartilage in the ventral side of trachea. (C) Transverse section of lung in (B) stained for Sox9. [file 1741-7007-11-117-S2.tiff]

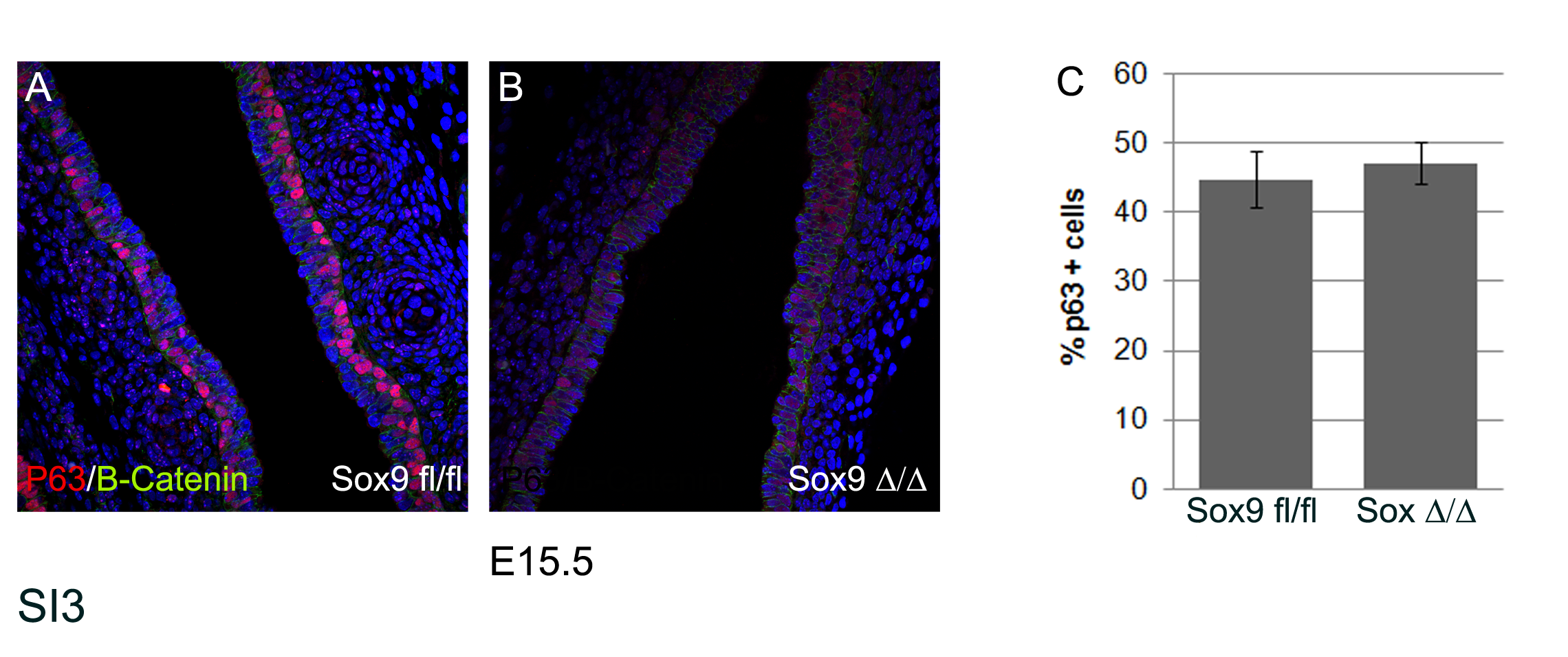

Supplement: Additional file 3: Figure S3 — Staining for P63 was used to determine changes in basal cells in E15.5 Sox9Δ/Δ trachea compared with Sox9fl/fl trachea. (A-C) Number of P63-positive cells in the tracheal epithelium was not affected by Sox9 deletion at embryonic day (E) 15.5. [file 1741-7007-11-117-S3.tiff]

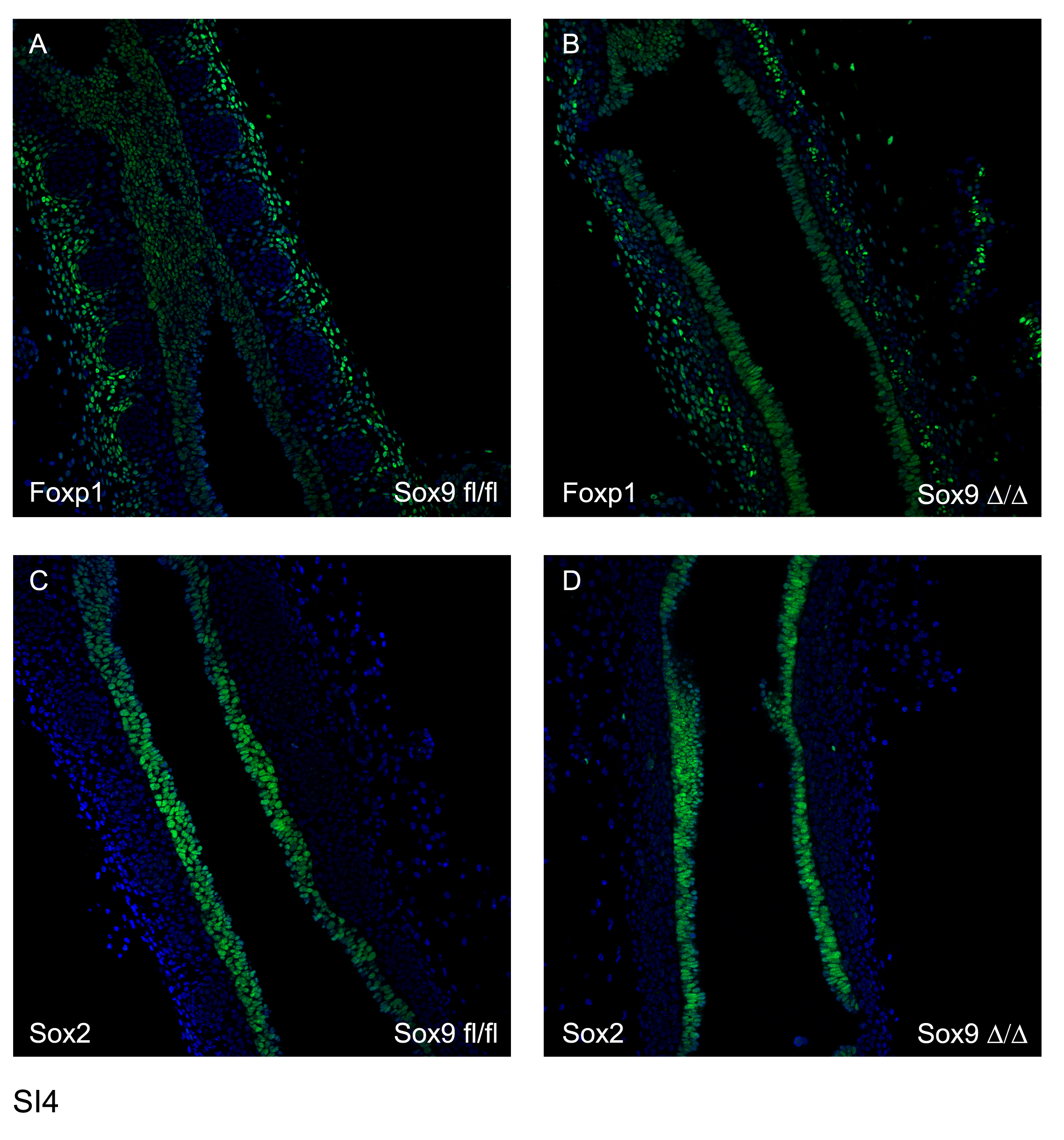

Supplement: Additional file 4: Figure S4 — Sox2 and Foxp1 expression was not altered in Sox9Δ/Δ tracheal epithelium. Immunofluorescence staining for Sox2 and Foxp1 did not show any qualitative or quantitative alterations of expression of these transcriptional factors in the mutant trachea. [file 1741-7007-11-117-S4.tiff]
